# Dynamics of plasma micronutrient concentrations and their correlation with serum proteins and thyroid hormones in patients with paracoccidioidomycosis

**DOI:** 10.1371/journal.pone.0226609

**Published:** 2019-12-26

**Authors:** Jeniffer Michelline de Oliveira Custódio, Iasmim Mayumi Enokida, Daniel Araujo Gonçalves, Sandra Maria do Valle Leone de Oliveira, James Venturini, Lidia Raquel Carvalho, Rinaldo Poncio Mendes, Anamaria Mello Miranda Paniago

**Affiliations:** 1 Postgraduate Course in Health and Development of the Central West Region, Federal University of Mato Grosso do Sul, Campo Grande, Mato Grosso do Sul, Brazil; 2 Scientific initiation CNPq, Faculty of Medicine - FAMED, Federal University of Mato Grosso do Sul, Campo Grande, Mato Grosso do Sul, Brazil; 3 Department of Chemistry, Minas Gerais State University - UEMG, Ituiutaba, Minas Gerais, Brazil; 4 Faculty of Medicine- FAMED, Center for Biological and Health Sciences- CCBS, Federal University of Mato Grosso do Sul, Mato Grosso do Sul, Campo Grande, Brazil; 5 Department of Biostatistics, Institute of Biosciences, State University Paulista "Júlio de Mesquita Filho" -UNESP, Botucatu, São Paulo Brazil; 6 Department of Tropical Diseases, Botucatu Medical School, Universidade Estadual Paulista (UNESP), Botucatu, São Paulo, Brazil; Universidade Federal do Espirito Santo, BRAZIL

## Abstract

Minerals, such as zinc, copper, and iron are reported to play roles in chronic infectious diseases; however, their role in paracoccidioidomycosis (PCM) remains unknown. This study aimed to examine the micronutrient dynamics and their correlation with serum proteins and thyroid hormones in patients with PCM. In 14 patients with PCM and 10 healthy subjects, we evaluated the body mass index (BMI) along with serum levels of hemoglobin, iron, ferritin, zinc, copper, magnesium, albumin, globulin, thyroid stimulating hormone (TSH), thyroxine (free T4), and triiodothyronine (T3). Evaluations were conducted at the first appointment, before treatment, and at the end of the first, second, fourth, and sixth month of PCM treatment. The control group was only evaluated once. We observed that before treatment, patients with PCM, had higher levels of copper and lower level of iron than those of the control group. After one month of treatment, the iron levels increased, whereas the levels of copper after six months of treatment. Reduction in inflammatory activity, indicated by the normalization of C-reactive protein, ferritin, albumin, and globulin levels, was observed during treatment. However, no correlation was observed between the serum levels of minerals and inflammatory activity or thyroid function in this study. In conclusion, our results showed higher serum copper levels in control group compared to those in pretreatment patients; the clinical importance of this observation should be investigated in further studies. After treatment, serum copper levels showed a tendency to decrease. In addition, serum iron levels were decreased at the stage of active disease, and were increased after treatment. Thus, serum iron levels can be used as a better biomarker for treatment control.

## Introduction

Paracoccidioidomycosis (PCM), caused by fungi of the genus *Paracoccidioides*, is a systemic mycosis endemic to Latin America. Similar to bacteria and humans, fungi require an adequate concentration of trace metals for sufficient cellular functioning, including respiration, replication, transcription, translation, and regulation of virulence [1, 2].

The requirement of trace elements for both the host and parasite, results in their competition for minerals. During most infections, the mechanisms by which defense cells control the invasion of microorganisms involve the redistribution of essential trace elements such as zinc, copper, and iron in serum, as well as increased synthesis of acute phase proteins such as ceruloplasmin. These changes are mediated by tumor necrosis factor α (TNF-α) as well as interleukin-1 (IL-1), and interleukin-6 (IL-6) [3].

Several systems have been developed by microorganisms to sequester metal ions from the host. Metal acquisition systems import and remove metals from extracellular sites using siderophores and acquisition of host proteins [1, 4, 5].

Although the importance of minerals such as zinc, copper, and iron for patients with chronic infectious diseases has been reported [1,6,7] there are no studies on the role of these elements in patients with PCM.

Other studies showed that *Paracoccidioides* spp. assimilates iron and zinc for development [4,8]. Zinc is likely to be associated with the catalytic agents that potentiate cell division and differentiation [2]. High concentrations of iron potentiate aconitase from *Paracoccidioides* spp. (pbACO), which is involved in parasite’s energy generation [9].

Involvement of the patient’s thyroid in response to *Paracoccidioides* spp., is very rare. However, functional alterations in the thyroid have been observed, with reduction of triiodothyronine (T3) and thyroxine (T4) levels, and normal to low levels of thyroid stimulating hormone (TSH) [10, 11]. These changes characterize the euthyroid sick syndrome, and have also been observed in other chronic infectious diseases, such as tuberculosis [12].

Hormonal disorders, present in patients with infectious and parasitic diseases (DIP), can be aggravated by the deficiency of antioxidant nutrients. This occurs when the hormonal action is directly associated with the intracellular oxidant/ antioxidant balance. Antioxidants are important for defense against oxidation, with selenium and zinc being the main antioxidant agents utilized by the thyroid [13].

Micronutrients strongly affect host-parasite interactions and these micronutrients are altered by inflammatory responses. Changes in the serum levels of micronutrients were assessed in patients with PCM, which is a disease with a chronic systemic inflammatory component and prolonged treatment. To the best of our knowledge, there are no studies that have evaluated the role of these trace elements in patients with PCM during the course of treatment. Therefore, we aimed to analyze the micronutrient dynamics in patients with PCM over the first six months of treatment, their relationships with inflammatory response and functional activity of the thyroid gland.

## Materials and methods

### Ethical aspects

This project was approved by the Human Research Ethics Committee of the Federal University of Mato Grosso do Sul (CEP—UFMS) (number 1.345.541). All the participants signed informed consent form.

### Location and period of study

This study was carried out between February 2016 and March 2017 on patients assisted at the Systemic Mycoses ambulatory care setting of the Infectious and Parasitic Diseases Unit of the Maria Aparecida Pedrossian University Hospital (UNIDIP-HUMAP) at the Federal University of Mato Grosso do Sul, a reference center for infectious diseases. Patients with PCM from the state of Mato Grosso do Sul are referred to this Service, independently of the degree of severity.

### Patients

#### Case definition

A patient was considered to have a confirmed case of PCM when presenting suggestive clinical manifestations and when typical forms of *Paracoccidioides* spp. were identified by direct mycological, culture, or histopathological examination using clinical samples, such as sputum, lymph node aspirate, oral mucosa scraping, and fragments of biopsied tissue.

#### Inclusion and exclusion criteria

All untreated patients with PCM at the site and during the study period were invited to participate in the study, and patients that met the following criteria were enrolled: active and confirmed PCM, under 60-years, and with no use of antifungal compounds.

Patients presenting another disease of neoplastic, inflammatory, or infectious origin, and/or using immunosuppressive medications were excluded. Smoking was not considered as an exclusion criterion.

#### Demographic, nutritional, and clinical data

Demographic data, clinical data, and nutritional evaluations were used for this study. The data were recorded by the same researcher during the first appointment and follow-up.

Demographic data related with sex and age were collected for the study.

The following clinical data were collected: weight before symptoms onset, at first appointment, and at follow-up; height; body mass index (BMI); smoker status; alcohol use; clinical form data; severity of the disease; organs or system involved; and the use of any antifungal compounds. Weight before symptoms onset was reported for every patient, but the weight and height were measured by a researcher on an anthropometric mechanical scale that was calibrated for accuracy before each use and in every clinical evaluation.

Normal BMI values were considered as 18.5 to 24.9 [14].

We defined smokers as those who reported smoking one or more cigarettes per day, whereas alcohol users were those who reported to be regularly drinking alcohol.

The daily intake of energy, iron, magnesium and zinc was determined by a 24 h recall was quantified as described by Pinheiro et al. 2008 [15] and Philippi 2016 [16]. Recommended Dietary Allowance (RDA), Estimated Average Requirement (EAR), or Adequate Intake (AI) were considered as reference values [17].

The clinical form of the disease was classified as acute/subacute or chronic. The acute/subacute form, also known as the juvenile form, is more frequent in children, adolescents and young adults, and the most affected organs are the lymph nodes, liver, spleen and bone marrow. The chronic or adult form is the most common form in patients older than 30 years of age, and affects the lungs and upper digestive and airway mucous membranes.

As to its severity, chronic form of PCM was subdivided as mild, moderate or severe. Mild cases were those with BMI loss lower than 5% of the patient´s usual BMI and involvement of a single or few organs or tissues without dysfunctions. Severe cases were defined by meeting three or more of the following criteria: a) BMI loss equal to or greater than 10%; b) intense pulmonary involvement; c) involvement of other organs, such as adrenal glands, central nervous system, and bones; d) presence of lymph nodes enlargement in multiple chains in superficial or deep, pseudotumoral form (>2.0cm in diameter, without suppuration) or suppurative form; e) high antibody titers. Cases were defined as moderate if they were intermediate between mild and severe forms.

Similarly, acute/subacute form were subdivided as moderate or severe. We considered moderate if the lymph nodes enlargement was inflammatory, non-suppurative; hepatomegaly/splenomegaly were absent or mild; BMI loss lower than 10%; and low antibody titers. On the other hand, severe cases were those with three or more of the following criteria: a) pseudotumoral or suppurative lymph nodes enlargement; b) BMI loss equal to or greater than 10%; c) hepatomegaly or splenomegaly present and intense; d) involvement of the other organs; e) high antibody titers [18].

Patients were treated with itraconazole or with the sulfamethoxazole-trimethoprim combination, cotrimoxazole (CMX), following the recommendations of the Brazilian Consensus on Paracoccidioidomycosis [19].

### Study design

This was an observational, prospective study, evaluating patients before treatment and during follow-up.

The sampling calculation was made considering a type I (alpha) error of 5% and a test power of 80%. Thus, the smallest number of individuals in each arm, the patients and controls, were calculated for each micronutrient as a function of the difference to be observed, as follows: a) copper: a difference of 0.27 and 7 individuals; b) zinc: a difference of 0.46 and 10 individuals; c) iron: a difference of 0.50 and 7 individuals; d) magnesium: a difference of 2.0 and 8 individuals. Based on these data and considering that micronutrient dosing was performed on the same samples, 14 patients and 10 healthy controls were studied.

The patients were evaluated for their serum or plasma levels of copper, zinc, iron, magnesium, hemoglobin, ferritin, albumin, globulins, TSH, T4L, and T3 before treatment, and in the first, second, fourth, and sixth month of treatment. This group of patients was divided into two subgroups: 13 patients with a chronic form of PCM (CF-PCM) and one patient with an acute/subacute form of PCM (ASF-PCM) [18].

The control group comprised 10 healthy male subjects, with no previous history of the diseases mentioned above, from the same region as the 14 PCM patients. They consisted of medical school staff and students. They were only admitted once for evaluating their serum or plasma levels of the aforementioned elements.

### Blood collection

Blood samples from the groups were obtained by venipuncture in siliconized vacuum polypropylene tubes, without anticoagulant to determine the metal content.

Blood samples for testing the iron, magnesium, albumin, globulins, C-reactive protein (CRP), hemoglobin, T3, free T4, and TSH levels, were also obtained by venipuncture in vacuum collection tubes with a separator gel for serology.

### Analysis procedures

#### Copper and zinc

Copper and zinc were analyzed in the Laboratory of Mineral Metabolism at the Federal University of Mato Grosso do Sul, according to the methodology of Ulbrecht et al. 2019 [20]. For quantifications, the samples were previously subjected to assisted digestion in a microwave oven to eliminate organic matter. Aliquots of 0.5 mL were digested using 1.00 mL HNO_3_ (65% Sigma-Aldrich), 1.00 mL H_2_O (18 MΩ cm, Milli-Q^®^, Millipore), and 0.50 mL H_2_O_2_ (30% m/m^-1^ Merck) in a microwave oven (Speedwave^®^ Four, Berghof Germany), equipped with a 12-position rotor. Samples were digested at 200°C for 40 min.

The elements were measured using an Inductively Coupled Plasma Optical Emission Spectrometer (ICP OES Thermo Scientific iCAP 6300). All determinations of Cu and Zn by ICP OES were performed in the axial view of plasma with a radio frequency power of 1250 W, sample flow rate of 0.45 L min^−1^, plasma gas flow rate of 12 L min^−1^, integration time of 15 s, stabilization time of 20 s, and nebulization pressure of 20 psi. The external calibration method was monitored at 324.7 nm for copper and at 213.9 nm for zinc. The calibration curves had a linear coefficient of 0.9999 and the detection and quantification limits were 0.0003 and 0.0008 for Cu and 0.0001 and 0.0004mgL^-1^ for Zn, respectively. Values considered normal by the manufacturer were 0.8 to 1.2 mg/L for copper and 0.7 to 1.2 mg/L for zinc.

#### Iron, magnesium, albumin, and globulins

Iron and magnesium, as well as albumin and globulin, were detected in the serum using colorimetric methods.

The values considered normal by the manufacturer were: 61 to 157 mcg/dL for iron, 1.27 to 2.6 mg/dL for magnesium, 3.5 to 5.5 g/dL for albumin, and 1.8 to 4.4 g/dL for globulins.

#### C-reactive protein (CRP)

Serum levels of high sensitivity CRP were measured using an immunoturbidimetric method. The values considered normal by the manufacturer were 0 to 5.0 mg/L.

#### Ferritin

The serum concentration of ferritin was determined using electrochemiluminescence. The values considered normal by the manufacturer were 30 to 400ng/mL.

#### Hemoglobin

Hemoglobin levels were evaluated using the automated cell counting instrument Sysmex XE2100 and was measured in units of hemoglobin. Values considered normal by the manufacturer were 13.5 to 17 g/dL.

#### T3, free T4, and TSH

The serum concentrations of T3, free T4, and TSH were determined using electrochemiluminescence.

According to the manufacturer, the normal TSH values ranged from 0.27 to 4.2 ng/mL, 0.93 to 1.7 ng/mL for free T4, and 0.8 to 2.0 ng/mL for T3.

### Statistical analysis

Categorical variables between groups were compared by Fisher’s exact test. Analysis of repeated measures was used to compare the mean values of numerical variables for the different periods during the treatment of patients with PCM. Friedman’s test was used to compare the median values of numerical variables of different periods during the patient’s treatment. The Mann-Whitney test was used to compare the median values between the control group and the PCM group. Correlations between variables were analyzed using the Pearson correlation test. A *p* value less than or equal to 0.05 was used to define the statistical differences.

Statistical tests were performed using SPSS.

## Results

**Fig 1:**
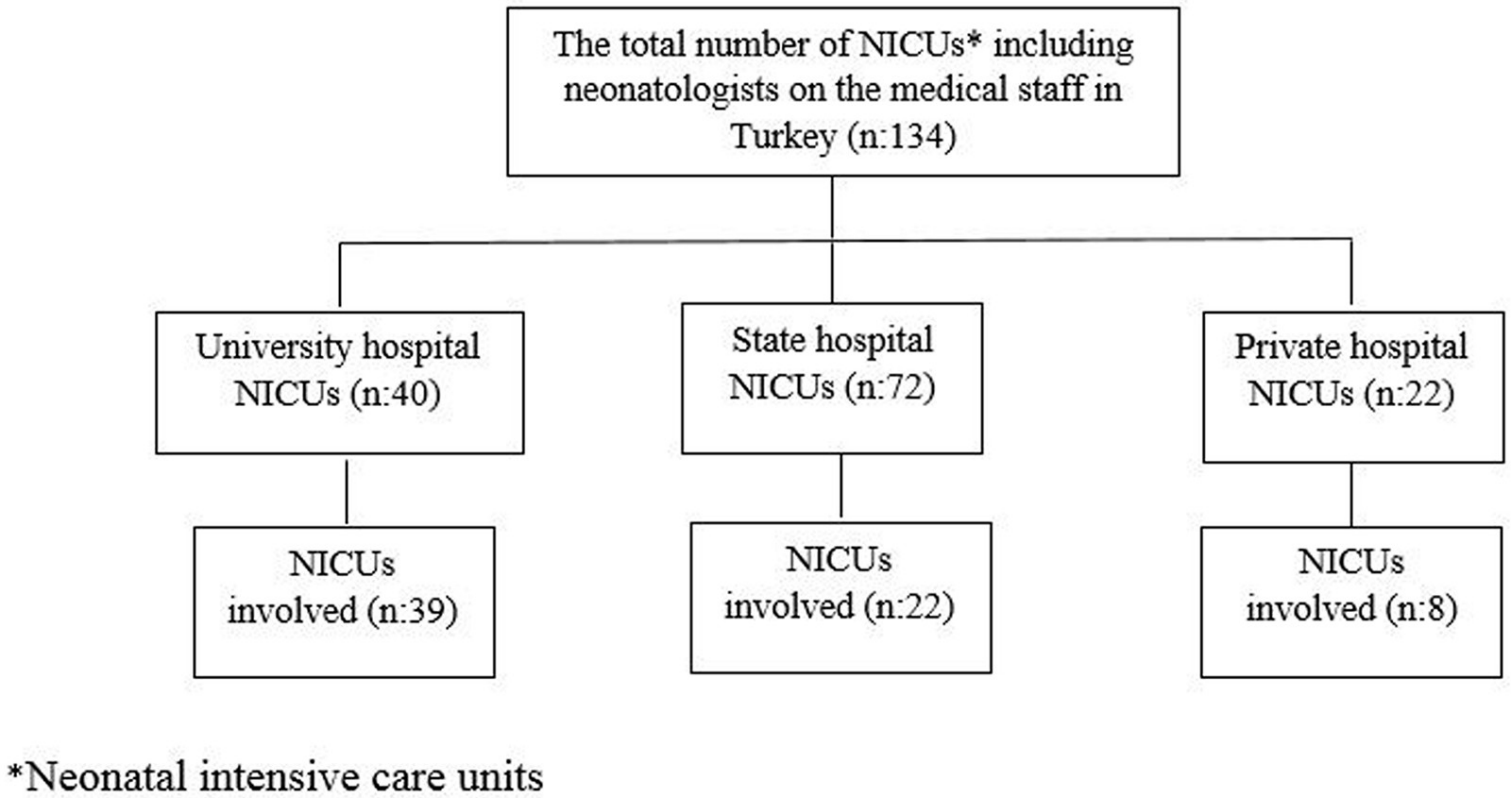
Flow diagram of participant selection and study design. Mo = months; CF-PCM-: chronic form of PCM; ASF-PCM: acute-subacute form of PCM.

As shown in Fig 1, 18 new PCM patients were reported to the hospital between February 2016 and March 2017.

### 1- Participant demographic, nutritional and clinical data

Table 1 shows the characteristics of PCM patients and shows no differences between patients treated with itraconazole or cotrimoxazole.

**Table 1:** Characteristics of 14 patients with paracoccidioidomycosis according to the antifungal compound used in their treatment.

Characteristics	PCM patients n = 14	Itraconazole n = 9	Cotrimoxazole n = 5	*P* Value*
	n (%) or Median [IQR]	n (%) or Median [IQR]	n (%) or Median [IQR]	
Age (years)	49.0 [46.8–56.0]	49.0 [48.0–-55.0]	49.0 [30.0–56.5]	0.74
Sex male	14 (100)	9 (100)	5 (100)	1.00
BMI reduction^#^	2.9 [1.8–4.1]	2.9 [2.0–4.4]	3.4 [0.5–3.9]	0.84
Clinical form/severity				
Mild chronic	2 (14.3)	1 (11.1)	1 (20.0)	1.00
Moderate chronic	8 (57.1)	7 (77.8)	1 (20.0)	0.09
Severe chronic	3 (21.4)	1 (11.1)	2 (40.0)	0.51
Severe Acute/Subacute	1 (7.2)	0	1 (20.0)	0.36
Organs involved ^##^				
Lungs	12 (85.7)	9 (100)	3 (60.0)	0.11
UADT	11 (78.6)	8 (88.9)	3 (60.0)	0.51
Skin	8 (57.1)	5 (55.6)	3 (60.0)	1.00
Lymph nodes	5 (35.7)	3 (33.3)	2 (40.0)	1.00
Adrenals	3 (21.4)	2 (22.2)	1 (20.0)	1.00
CNS	1 (7.1)	0	1 (20.0)	1.00

IQR: interquartile range, BMI: body mass index in Kg/m^2^, UADT: upper air digestive tract, CNS: Central Nervous System.

^#^ Between onset of symptoms and PCM diagnosis.

^##^ One patient could have more than one affected organ

* Mann Whitney U test for the comparison of numerical variables and Fisher for comparison of categorical variables.

The demographic, nutritional and clinical characteristics of all participants are shown in Table 2. The PCM and control groups did not differ in age, sex, alcohol use, or daily micronutrient intake. The PCM patient group had a lower median BMI, higher frequency of smoking and higher number of rural workers than those in the control group.

**Table 2:** Characteristics of 14 patients with paracoccidioidomycosis (PCM group) and a control group of 10 healthy subjects.

Characteristics	PCM group n = 14	Control group n = 10	*P* value *
	n (%) or Median [IQR]	n (%) or Median [IQR]	
Age (years)	49.0 [46.8–56.0]	42.5 [30.5–54.0]	0.31
Sex male	14 (100)	10 (100)	1.00
BMI (kg/m^2^)	21.0 [18.6–23.7]	27.5 [23.1–28.7]	<0.01
Energy (kcal/day)	1632.3 [1286.4–1968.2]	1888.5 [1540.1–2315.6]	0.21
Iron ^#^	15.1 [10.8–20.3]	15.1 [9.7–16.9]	0.70
Magnesium ^#^	192.4 [119.4–265.5]	195.0 [149.3–255.4]	0.75
Zinc^#^	9.1 [5.2–14.7]	10.0 [7.1–14.2]	0.84
Copper^#^	0.67 [0.5–1.6]	0.57 [0.6–1.0]	0.75
Rural worker	12 (85.7)	0 (0)	<0.01
Cigarette smoking	12 (85.7)	0 (0)	<0.01
Alcohol use	6 (28.6)	1 (0)	0.12

IQR: interquartile range, BMI: body mass index.

* Mann Whitney U test for the comparison of numerical values and Fisher´s exact test for comparison of categorical variables.

^#^ daily intake in mg

### 2- Concentration of inflammatory proteins, hemoglobin, and trace elements as well as TSH, T3, and free T4 in patients with paracoccidioidomycosis before treatment and in the control group

Before treatment, the group of patients with PCM had higher levels of copper, CRP, and globulins,and lower levels of iron, hemoglobin, and albumin than those of the control group. The mean zinc levels were low in both groups (Table 3).

**Table 3:** Serum levels of minerals, proteins and thyroid hormones from 14 patients with paracoccidioidomycosis (PCM group) before treatment and from 10 healthy subjects (control group).

Variables	PCM Group (n = 14) Mean (SD) or median [IQR]	Control Group (n = 10) Mean (SD) or median [IQR]	*P* Value
Magnesium (mg/dL)	1.87 (0.32)	2.09 (0.12)	0.07
Zinc (mg/L)	0.24 (0.32)	0.55 (0.38)	0.20
Copper (mg/L)	1.16 (0.40)	0.58 (0.17)	<0.01
Iron (μg/dL)	52.30 (25.97)	116.29 (34.08)	<0.01
Hemoglobin (g/dL)	13.16 (2.80)	15.84 (0.73)	0.01
Ferritin (ng/mL)	511.88 (357.43)	264.08 (183.93)	0.09
Albumin (g/dL)	3.98 (0.80)	4.74 (0.15)	0.01
Globulin (g/dL)	3.56 (0.66)	2.58 (0.88)	0.01
C-ReactiveProtein (mg/dL)	40.59 [9.89–71.12]	0.97 [0.76–2.39]	<0.01*
TSH (ng/mL)	2.69 (1.85)	2.23 (1.58)	0.54
Free T4 (ng/mL)	1.23 (0.22)	1.31 (0.27)	0.46
T3 (ng/mL)	1.12 (0.31)	1.32 (0.18)	0.13

IQR: interquartile range, SD: standard deviation TSH: thyroid stimulating hormone, T3: triiodothyronine, T4: thyroxine

Student’s *t*-test for comparing means and Mann-Whitney * test for comparison of medians

### 3- Concentration of inflammatory proteins, hemoglobin, and trace elements as well as TSH, T3, and free T4 in patients with paracoccidioidomycosis before and during the PCM treatment

Changes were noted in some of the variables studied during the PCM treatment (Table 4). A rise in BMI was observed in the first and second months and it then remained stable in the subsequent months of the study. Albumin rose in the first month and then remained stable in the following months. Ferritin and CRP decreased in the first month of treatment, whereas globulin reduction was observed after the second month of treatment (Table 4).

**Table 4:** Serum levels of minerals, proteins, thyroid hormones and body mass index (BMI) of 14 patients with paracoccidioidomycosis (PCM group) before and during PCM treatment.

Variables	Follow-up Mean (SD) or Median [IRQ]					
	pretreatment	1mo	2mo	4mo	6mo	*P*
Magnesium (mg/dL)	1.87 (0.32)	2.33(1.24)	1.91 (0.28)	1.99 (0.24)	2.00 (0.19)	0.30
Zinc (mg/L)	0.31 (0.33)	0.75 (0.37)	0.64 (0.29)	0.69 (0.33)	0.61 (0.32)	0.09
Copper (mg/L)	1.16 (0.40)	0.99 (0.21)	0.96 (0.27)	0.92 (0.20)	0.85 (0.23)	0.08
Iron (μg/dL)	52.3 (26.0)^b^	80.2 (23.4)^a^	92.3 (28.0)^a^	85.1 (22.5)^a^	82.5 (21.5)^a^	<0.01
Hemoglobin (g/dL)	13.2 (3.1)^c^	13.8 (1.8)^bc^	14.4 (1.6)^ba^	15.0 (1.6)^a^	15.2 (1.6)^a^	<0.01
Ferritin (ng/dL)	511.9 (357.4)^a^	391.3 (275.5)^b^	313.5 (236.4)^bc^	309.6 (247.3)^bc^	279.3 (165.4)^c^	<0.01
Albumin (g/dL)	4.0 (0.8)^b^	4.3 (0.4)^a^	4.4 (0.2)^a^	4.6 (0.3)^a^	4.5 (0.4)^a^	0.01
Globulin (g/dL)	3.6 (0.7)^a^	3.3 (0.6)^ab^	3.2 (0.5)^b^	2.7 (0.5)^c^	2.7 (0.4)^c^	<0.01
CRP (mg/dL)	40.1[8.6–58.4]^a^	4.8 [3.7–10.8]^b^	5.1 [2.4–8.0]^b^	3.2 [1.4–6.2]^b^	2.7 [1.2–6.1]^b^	0.02*
BMI (kg/m^2^)	21.2 (3.7)^c^	22.1 (3.6)^b^	23.0 (4.0)^a^	22.9(3.9)^a^	22.6 (3.8)^a^	<0.01
TSH (ng/mL)	2.7 (1.9)	2.3 (1.2)	3.0 (2.0)	3.2 (2.0)	2.4 (1.1)	0.66
Free T4 (ng/dL)	1.2 (0.2)	1.1 (0.2)	1.1 (0.2)	1.1 (0.2)	1.2 (0.2)	0.24
T3 (ng/mL)	1.1 (0.3)	1.5 (0.8)	1.2 (0.1)	1.2 (0.3)	1.7 (1.3)	0.25

IQR: interquartile range, CRP: C reactive protein, BMI: Body mass index, Mo: months. TSH: thyroid stimulating hormone, T3: triiodothyronine, T4: thyroxine

Values followed by the same letter are not significantly different (p > 0.05) and values followed by different letters present significant differences (p ≤ 0.05). The letter "a" indicates the highest value. *P* values were determined by repeated measures analysis, except for the CRP variable that was determined using Friedman’s test *.

Of the oligoelements, the only one that changed during the first six months of treatment was iron, which showed an increase after one month and then reached normal stable levels. Magnesium, zinc, and copper levels remained unchanged over the first six months of treatment (Table 3).

Mean values of TSH, free T4, and T3 levels were normal before treatment and did not change significantly during treatment (Table 3). Before treatment, only the patient with a severe acute-subacute form of PCM had low T3 and high TSH levels.

Serum or plasma levels of micronutrients, proteins, and thyroid hormones did not differ before and during follow-up when compared with those of patients treated with itraconazole and cotrimoxazole (S1 and S2 Tables).

Among the patients, the only direct correlation was observed between blood plasma and serum levels of copper and magnesium. In the control group, direct correlations were observed between magnesium and T3, and copper and zinc levels, and a tendency for direct correlation was observed between BMI and T4L (p = 0.058), as shown in Table 5.

**Table 5:** Correlation between serum levels of minerals, proteins, and thyroid hormones and body mass index (BMI) in 14 patients with paracoccidioidomycosis (PCM group) before treatment (A) and in 10 healthy subjects (control group) (B).

**A**	**PCM Group**								
		**BMI**	**TSH**	**T4L**	**T3**	**Cu**	**Zn**	**Mg**	**Fe**
TSH	R	-0.365							
	P	0.200							
T4	R	0.272	-0.219						
	P	0.347	0.452						
T3	R	0.388	-0.372	0.029					
	P	0.212	0.234	0.928					
Cu	R	-0.266	0.327	0.474	0.064				
	P	0.358	0.254	0.087	0.843				
Zn	R	-0.008	0.088	0.288	-0.065	0.348			
	P	0.978	0.765	0.318	0.841	0.223			
Mg	R	0.031	0.180	0.435	0.347	**0.735**	0.403		
	P	0.916	0.538	0.120	0.270	**0.003**	0.153		
Fe	R	-0.253	-0.186	0.223	0.373	-0.100	0.111	0.180	
	P	0.384	0.523	0.443	0.232	0.735	0.705	0.538	
PCR	R	-0.457	0.240	0.070	-0.498	0.374	0.106	0.289	-0.411
	P	0.135	0.452	0.828	0.119	0.231	0.743	0.363	0.184
**B**	**Control Group**								
		**BMI**	**TSH**	**T4L**	**T3**	**Cu**	**Zn**	**Mg**	**Fe**
TSH	R	0.440							
	P	0.236							
T4L	R	**-0.651**	-0.499						
	P	**0.058**	0.171						
T3	R	0.497	0.089	0.208					
	P	0.256	0.850	0.655					
Cu	R	0.060	-0.253	0.234	0.635				
	P	0.869	0.511	0.545	0.126				
Zn	R	-0.514	-0.065	0.549	0.524	0.621			
	P	0.128	0.868	0.125	0.228	0.055			
Mg	R	-0.150	0.074	0.231	**0.748**	**0.002**	**0.838**		
	P	0.679	0.850	0.551	**0.050**	**0.007**	**0.002**		
Fe	R	0.177	0.528	0.082	-0.100	-0.023	0.073	-0.087	
	P	0.625	0.144	0.834	0.832	0.950	0.842	0.811	
CRP	R	0.045	-0.261	-0.519	-0.275	0.130	-0.133	-0.163	0.117
	P	0.924	0.571	0.233	0.551	0.781	0.777	0.727	0.802

For units of measurement see Table 4

BMI: body mass index, TSH: thyroid stimulating hormone, T3: triiodothyronine, T4: thyroxine, Cu: copper, Zn: zinc, Mg: magnesium, Fe: Iron, CRP: C-reactive protein.

PCM group = Significant correlation between Mg and Cu (r = 0.735, p = 0.003).

Control group = Significant correlation between Mg and T3 (r = 0.748, p = 0.05), Mg and Cu (r = 0.789, p = 0.007), Mg and Zn (r = 0.838, p = 0.002)., tendency for direct correlation was observed between BMI and T4L (r = - 0.651, p = 0.058).

## Discussion

Nutritional status is a major modulator of the immune response and both macronutrient and micronutrient deficiencies can contribute to an ineffective host response [21]. The present study, performed in a representative sample of PCM patients, found a significant improvement in nutritional status with the application of specific treatment. Although the BMI of patients upon first appointment could be considered normal, 71.4% of them presented a weight loss of more than 10% in their body weight. This could be associated with the action of hormones, cytokines that decrease appetite, and a high catabolism observed in the infected individuals. Furthermore, this large weight loss is one of the criteria used to diagnose PCM infection [18]. Dietary supplementation with nutrients may contribute to the restoration of immune function and therefore resistance to *Paracoccidioides* infection.

Low levels of albumin and elevated levels of globulins have been found in studies on patients with PCM [22, 23]. Marquez et al. [24] analyzed the blood serum proteins of 30 patients with chronic PCM and 12 patients with acute PCM, and found high concentrations of globulins in both groups. The present study revealed elevated globulin levels in patients, which progressively decreased after starting treatment, particularly by the fourth month, thus confirming the results of previous studies [24, 25]. Lower levels of albumin were also identified in PCM patients before treatment, and an increase was observed in the first month of treatment. Reduction in blood serum albumin can be explained by the increased consumption of albumin caused in the infectious state and the deviations of the metabolic pathway, which require an increase in the production of other proteins during the inflammatory process [25, 26].

Changes in the plasma concentrations of the CRP, which is considered a marker of inflammation, are commonly reported in people with infectious diseases, especially during an inflammatory response [27]. In the present study, CRP levels were high, this finding was similar to those of other studies conducted in Korea and Brazil on patients with tuberculosis [28, 29]. Studies on patients with tuberculosis have demonstrated that CRP aid in the diagnosis of TB and also act as a marker of smear-positive persistence at one month after treatment [28]. However, treatment of PCM lasts longer than that of tuberculosis, which could explain the persistence of high CRP levels even after six months of treatment. These observations indicate the presence of inflammatory activity in the patients involved in this study and could be useful in the development of a treatment [18].

In general, micronutrient deficiency suppresses the functions of immune responses by affecting the host innate immunity and deregulating the polarization of T cell-mediated adaptive immune responses and humoral immunity [30]. In chronic infectious diseases, such as American tegumentary leishmaniasis, copper, iron, zinc, and selenium all participate in various stages of phagocyte proliferation and maturation; they also support the cytotoxic capacity of these phagocytes and trigger a Th1 type immune response, which is important for protecting the host against infection [31, 32]. In addition, studies conducted on tuberculosis patients showed that the serum concentrations of trace elements, could change and are influenced by the diet and physiology of the host or pathogen [28, 33].

Alternately, similar findings to those from our study have been reported, including detection of high serum levels of copper during the initial phase of other chronic infectious diseases [31, 33]. Copper ions have been observed to be crucial for the control of intracellular bacteria by optimizing their oxidative attack on *Mycobacterium tuberculosis* within macrophagic phagolysosomes [34]. Thus, elevated levels could indicate an attempt by the patient’s immune system to control *Paracoccidioides* spp. infection. However, these ions have also been implicated in the canonical activation of NRLP3 inflammasomes and consequent production of the proinflammatory cytokine IL-1β [35]. Elevated production of hydrogen peroxide and IL-1β by mononuclear phagocytes and an increased expression of NRLP3 have also been observed in patients with a chronic form of PCM before the start of antifungal treatment [36, 37]. This could explain the observed imbalance in the inflammatory responses of patients with PCM in the present study, in which a more deleterious than protective inflammatory profile prevailed, with copper playing an important role in this process.

In this study, no difference was observed in plasma zinc levels between patients with PCM before treatment and the control subjects. Both groups presented low daily intake and low plasma zinc levels, but the concentration of zinc tended to increase during treatment. For the majority of individuals, consumption of zinc in the Brazilian diet is at borderline low levels, and is at low levels in certain groups such as the elderly and children [38, 39]. Studies on patients with pulmonary tuberculosis [40] and visceral leishmaniasis [41] have revealed low amounts of zinc in the active phase of these diseases.

Zinc deficiency has been associated with impaired immunity [42,43] and has also been directly linked to the death of intracellular pathogens [44,45]. Curcio et al. [2] evaluated the regulation of zinc by *Paracoccidioides lutzii* membrane proteins, and found that zinc deprivation can elevate cell stress, decrease glucose uptake and in turn limit the energy generation required for infection by this pathogen. These changes have repercussions on cell wall formation and interfere with the success of infection. Therefore, Curcio et al. [2] suggested that the use of zinc chelators could positively contribute to the treatment of PCM.

Serum iron levels of the patients, in the present study, were lower than those in the control group. Iron is a ubiquitous nutrient in living cells, including those of humans, plants, and animals. Microorganisms, such as fungi and bacteria, also need this mineral in substantial amounts to multiply in the host. The need for this nutrient in both humans and pathogens results in competition for iron. This involves the iron import systems of microorganisms competing against their systems of withdrawal and sequestration of iron by macrophages, in which both compete for iron at the parasite-host interface [46, 47]. One of the main mechanisms of iron removal in the body is by increasing the levels of ceruloplasmin to oxidize iron and facilitate its transport by ferritin to different tissues. This creates a scarce iron environment to prevent the growth and expansion of pathogen. For iron uptake by microorganism, the mechanisms involve the import of metal, acquisition of host proteins, and removal of the metals from extracellular sites by siderophores; the latter is considered the main route by which most pathogens can extract iron from the host [1, 4, 5]. All these mechanisms can be potentiated when the pathogen is deprived of iron during its growth [6]. In the present study, copper levels were higher in patients with PCM than those in the control group, which may be related to the increased production of ceruloplasmin, preventing the use of iron by the parasite. In addition, ferritin values were also higher prior to the treatment. Reuse of iron in patients with chronic infectious diseases could be reduced because of the formation of iron deposits. This blockage was caused by increased IL-1 production in defense cells, such as neutrophils and macrophages. This clinical entity can be defined as anemia of chronic disease (ACD) [48].

Magnesium participates in several biological functions, acting as a cofactor for more than one hundred enzymatic reactions and for regulating the host immune response [49]. Normal levels of magnesium were identified in the patients of this study. The increase in magnesium occurs when there is a reduction in plasma levels of zinc in patients in the acute and chronic stages of leishmaniasis [35]. However, these findings were not confirmed in the present study on patients with chronic PCM. In our study, we observed a tendency for zinc to increase during treatment, but before treatment these values did not differ from the control group. A positive correlation was observed between copper and magnesium levels in the patients of this study. Both micronutrients affect the maturation of lymphoid tissues and cells and the reduction of nitric oxide (NO) in macrophages that are responsible for the release of free radicals in the body. The benefits of magnesium in relation to its action against macrophages are controversial, because some researchers believe that the effects of magnesium on NO may impair microbicide activity. Murray et al [50] identified NO as a leishmanicidal agent. The decreased zinc and increased magnesium in patients with leishmaniasis seemed to increase their susceptibility to the infection [41].

CRP levels might cause changes in the roles of micronutrients [51]. High concentrations of CRP are usually seen in patients with a low plasma zinc concentration and high serum copper levels [52]. In the present study, no correlation was observed between CRP and serum or plasma levels of minerals.

Several infectious and parasitic diseases can affect thyroid function and cause various clinical manifestations. *Paracoccidioides* spp. have tropism for various organs including the endocrine glands, adrenal gland and, less frequently the thyroid gland [11, 53–56]. In 1988, Kiy et al. [10] evaluated the baseline and post-test plasma thyrotropin releasing hormone (TRH) levels of T3 and T4 hormones in patients with PCM and detected low levels of T3 in patients with the acute form of the disease and in those with the severely disseminated chronic form. Brandão et al. found low levels of T3 in 47% of the patients who participated in the study [11]. In our study, we found no difference in free T4, T3, or TSH in patients with PCM compared to the control group, and no changes were observed during treatment. Two of the 14 patients, who had low levels of T3 and slightly elevated levels of TSH, had the acute/subacute clinical and the severe chronic form of the disease, respectively. It is possible that the decreased serum T3 levels with no or few changes in T4 and TSH as observed in PCM, in this and other studies [10,11], may be associated with disease severity, as it was also reported in tuberculosis [12]. In euthyroid patient syndrome, alterations inT3 are not due to a thyroid disease, but due to mechanisms that are not completely understood involving a decrease in the peripheral conversion of T4 to T3. In addition, the increase in proinflammatory cytokines, which are important mediators of the acute phase of illness but with inhibitory effects on the peripheral thyroid hormone metabolism, could play an important role in disease pathogenesis [57].

Our study has some limitations. First, the sample size was small. However, it is important to consider that the low incidence of PCM (about 17 new cases per year) [58] makes it difficult to obtain a large sample with new cases that meet the study criteria at a single center. We hope that publication of our results will contribute to future studies, including systematic reviews on the issue. Secondly, the control group comprised of non-smoking individuals, while there was a high prevalence of smoking among patients with PCM. Smoking is associated with higher levels of copper in males [59], which may have played a role in the copper levels observed in the patient group. Serum iron levels seem to be the least affected by smoking [60]. Another aspect to consider is that the control group consisted of medical school staff and students and not rural workers. The different epidemiological factors between the studied groups, including the nutritional factors, could influence the results. However, it should be considered that similar daily intakes of the analyzed nutrients were demonstrated for both groups. In conclusion, we observed that patients with PCM have higher serum copper and lower serum iron levels than the control group before treatment; however, the iron levels increased after one month of treatment, whereas the copper levels did not decrease after six months of treatment. A reduction in inflammatory activity, caused by the normalization of CRP, ferritin, albumin, and globulin levels, was observed during treatment. However, no correlation between the serum levels of minerals and inflammatory activity or thyroid function was observed in this study.

Knowledge about host and pathogen pathway modulations, in relation to micronutrients, contributes to the creation of medical strategies aimed at controlling the access of pathogens to metals and thereby decreasing their ability to cause infection.

## Supporting information

S1 Table Serum or plasma levels of micronutrients, proteins and thyroid hormones before and during PCM treatment according the antifungal compound. (DOCX)

S2 Table Serum or plasma levels of iron and C reactive protein before and during PCM treatment, according the antifungal compound. (DOCX)

S1 Dataset Dataset containing variables of the study. (XLSX)
