# Correction: Dynamics of plasma micronutrient concentrations and their correlation with serum proteins and thyroid hormones in patients with paracoccidioidomycosis

**DOI:** 10.1371/journal.pone.0228386

**Published:** 2020-01-24

**Authors:** The PLOS ONE Staff

The second author's name is spelled incorrectly. The correct name is: Iasmin Mayumi Enokida.

Fig 1 is incorrect. Please see the correct Fig 1 here. The publisher apologizes for the error.

**Fig 1:**
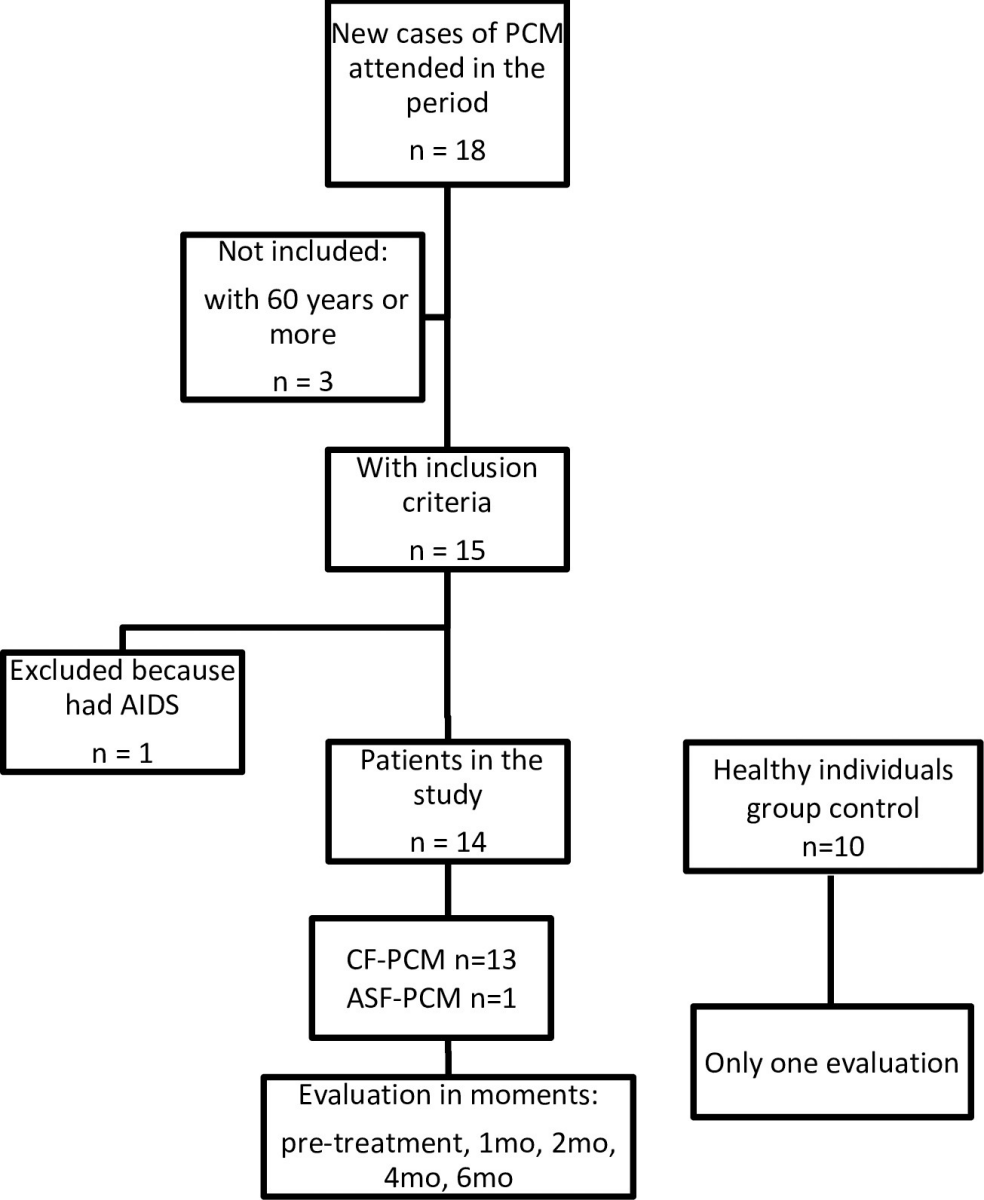
Flow diagram of participant selection and study design. Mo = months; CF-PCM-: chronic form of PCM; ASF-PCM: acute-subacute form of PCM.
