# Supplementary material for: Dynamics of plasma micronutrient concentrations and their correlation with serum proteins and thyroid hormones in patients with paracoccidioidomycosis
Source: PLoS One. 2019 Dec 26;14(12):e0226609. doi: 10.1371/journal.pone.0226609 (PMC6932777; doi:10.1371/journal.pone.0226609)
Supplement: S1 Table — (DOCX) [file pone.0226609.s001.docx]

**S1 Table.** Serum or plasma levels of micronutrients, proteins and thyroid hormones before and during PCM treatment according the antifungal compound.

| **Variables** | **Follow-up**  **Mean (SD)** | | | | |  |
| --- | --- | --- | --- | --- | --- | --- |
| antifungal | pretreatment | 1 month | 2 months | 4 months | 6 months | *P^*^* |
| **BMI (kg/m^2^)** |  |  |  |  |  |  |
| cotrimoxazole | 18.7 (3.3) | 20.0 (2.2) | 20.9 (2.4) | 21.3 (1.9) | 21.5 (1.9) | 0.10 |
| itraconazole | 22.5 (3.4) | 23.2 (3.9) | 24.2 (4.3) | 21.3 (9.0) | 23.4 (4.7) |  |
| *P*** | 0.22 |  |  |  |  |  |
| **TSH (ng/mL)** |  |  |  |  |  |  |
| cotrimoxazole | 3.4 (2.7) | 1.9 (0.9) | 3.2 (2.8) | 2.3 (1.1) | 2.0 (1.0) | 0.56 |
| itraconazole | 2.3 (1.2) | 2.6 (1.3) | 2.9 (1.6) | 3.9 (2.3) | 2.6 (1.2) |  |
| *P*** | 0.96 |  |  |  |  |  |
| **Free T4 (ng/mL)** |  |  |  |  |  |  |
| cotrimoxazole | 1.2 (0.2) | 1.1 (0.2) | 1.0 (0.2) | 1.0 (0.1) | 1.3 (0.3) | 0.07 |
| itraconazole | 1.2 (0.3) | 1.2 (0.2) | 1.1 (0.2) | 1.2 (0.2) | 1.2 (0.1) |  |
| *P*** | 0.60 |  |  |  |  |  |
| **T3 (ng/mL)** |  |  |  |  |  |  |
| cotrimoxazole | 0.9 (0.3) | 1.9 (1.4) | 1.2 (0.1) | 1.2 (0.2) | 1.9 (1.7) | 0.97 |
| itraconazole | 1.2 (0.3) | 1.3 (0.3) | 1.2 (0.1) | 1.2 (0.3) | 1.5 (1.1) |  |
| *P*** | 0.14 |  |  |  |  |  |
| **Copper (mg/dL)** |  |  |  |  |  |  |
| cotrimoxazole | 1.2 (0.6) | 0.9 (0.2) | 1.0 (0.2) | 0.9 (0.2) | 0.8 (0.2) | 0.23 |
| itraconazole | 1.1 (0.3) | 1.0 (0.2) | 0.9 (0.3) | 0.8 (0.4) | 0.9 (0.2) |  |
| *P*** | 0.81 |  |  |  |  |  |
| **Zinc (mg/dL)** |  |  |  |  |  |  |
| cotrimoxazole | 0.1 (0.1) | 0.6 (0.3) | 0.6 (0.3) | 0.7 (0.1) | 0.6 (0.3) | 0.06 |
| itraconazole | 0.3 (0.4) | 0.8 (0.4) | 0.6 (0.3) | 0.7 (0.4) | 0.6 (0.3) |  |
| *P*** | 0.49 |  |  |  |  |  |
| **Magnesium (mg/dL)** |  |  |  |  |  |  |
| cotrimoxazole | 1.7 (0.2) | 2.7 (2.2) | 1.7 (0.3) | 1.8 (0.2) | 2.0 (0.2) | 0.13 |
| itraconazole | 2.0 (0.3) | 2.1 (0.1) | 2.1 (0.2) | 2.1 (0.2) | 2.0 (0.2) |  |
| *P*** | 0.22 |  |  |  |  |  |

BMI: Body mass index, TSH: thyroid stimulating hormone, T3: triiodothyronine, T4: thyroxine

* *P* values for the follow-up comparison and ** *P* values for the antifungal comparison (Profile Analysis).
