# Supplementary material for: Dynamics of plasma micronutrient concentrations and their correlation with serum proteins and thyroid hormones in patients with paracoccidioidomycosis
Source: PLoS One. 2019 Dec 26;14(12):e0226609. doi: 10.1371/journal.pone.0226609 (PMC6932777; doi:10.1371/journal.pone.0226609)
Supplement: S2 Table — (DOCX) [file pone.0226609.s002.docx]

**S2 Table.** Serum or plasma levels of iron and C reactive protein before and during PCM treatment, according the antifungal compound.

| **Variable** | Follow up  Median [IQR] | | | | |  |
| --- | --- | --- | --- | --- | --- | --- |
| Antifungal | Admission | 1 Month | 2 Months | 4 Months | 6 Months | *P** |
| **Iron** |  |  |  |  |  |  |
| cotrimoxazole | 48.0 [33.5-60.5] | 84.0 [49.0-85.8] | 87.0 [71.8-99.3] | 73.0 [62.3-116.8] | 90.0 [78.8-101.9] | 0.08 |
| itraconazole | 56.0 [27.0-78.0] | 89.0 [74.0-91.5] | 76.0 [66.5-116.8] | 84.0 [68.4-93.0] | 73.0 [22.0-87.5] | 0.10 |
| *P*** | 0.79 | 0.34 | 0.95 | 0.88 | 0.17 |  |
| **CRP** |  |  |  |  |  |  |
| cotrimoxazole | 41.0 [13.7;155.1] | 6.0 [4.3;33.3] | 3.6 [2.2;6.2] | 1.7 [1.1;6.6] | 1.7 [1.2;28.0] | 0.17 |
| itraconazole | 40.1 [14.9;50.5] | 4.2 [2.1;8.4] | 6.6 [2.0;9.3] | 5.3 [2.2;6.6] | 3.6 [2.7;6.8] | 0.33 |
| *P*** | 0.76 | 0.20 | 0.72 | 0.43 | 0.53 |  |

IQR- interquartile range; CRP – C-reactive protein

* *P* values for the follow up comparison (Friedman Test) and ** *P* values for the antifungal comparison (Mann-Whitney U test).
